# Hints for Travellers in the near East

**Published:** 1903-06-13

**Authors:** 


### EDITOR'S LETTER-BOX.

**[Our Correspondents are reminded that prolixity is a great bar to publication, and that brevity of style and conciseness of statement greatly facilitate early insertion.]**

### HINTS FOR TRAVELLERS IN THE NEAR EAST.

Mr. E. A. REYNOLDS-BALL writes from 27 Chancery Lane, W.C.:—

I regret to be obliged to take serious objection to certain statements in a review of my "Hints for Travellers in the Near East," which appeared in your issue of the 23rd ult. The statements I complain of are as follows:—"An important feature of this little work is the care with which exactly the proper articles for 'travellers in the near East' to purchase before starting on their adventures are indicated: and it doubtless will not escape the notice of such travellers that in the advertisement pages are given full particulars of each one of the articles recommended in the text by the author." Now I think it is indisputable that the only inference the average reader would draw from this is practically that the recommendations of certain articles are not *bona fide*, but are influenced by the advertisements. The statement seems further to indicate that only articles which are advertised are mentioned in the text. I must maintain that these are flagrant examples of *suppressio veri*, and *suggestio falsi*, and are calculated to damage the sale of the work, while they seriously reflect upon my reputation as a writer. I must then ask for some apology for these very disparaging observations.

It is perhaps not the place here to demonstrate fully the inaccuracy of the conclusions arrived at by your reviewer, but I may just state:—1. That many articles are recommended in the text which not only are not advertised, but for which no advertisement was applied for. 2. I must assert most emphatically that the recommendation of any particular article is not influenced by the fact that an advertisement of this article appears in the advertisement pages. The attitude I have invariably taken up with regard to the question of recommendation of advertised articles is clearly shown in the following extract from a letter to a large advertiser:—"I much regret that I could not undertake to submit anything I say in the text about your machine for your approval. To do this would be tantamount to an editorial notice being influenced by an advertisement. The strong point of all my guide books (*vide* review of the *Scotsman*) is their impartiality and independence, in which I may be pardoned for saying that they differ from most publications of this kind. All I should propose to do would be to give a very short and guarded incidental reference to —— being eminently suitable for travellers on account of its extreme portability, handiness, and strength. But the actual wording must be left to my absolute discretion. Regarding the matter purely from the advertiser's point of view, I should consider that such brief but judiciously worded and obviously dispassionate reference would have far more weight with the public than a fulsome panegyric attributing to the —— merits superior to those of every other —— in existence irrespective of price." A reference, but not necessarily a recommendation to an article which is advertised is, no doubt, nearly always given, but no one can, I think, take exception to this, especially as my advertisement agents were instructed only to apply to firms whose articles I considered were worthy of being referred to in the text. As for anything approaching to a recommendation in the text my agents were expressly instructed to say that I could guarantee no recommendation (beyond the mere reference), but that this must be left solely to my discretion. 3. Finally I may add that in all my guide books trustworthiness, independence, and absolute freedom from what may be described as advertisement influence are their most noteworthy features. Indeed, this independence of attitude as regards advertisers is expressly pointed out in the following review from the *Scotsman*, of which I give an extract: "Deals with many material points, from a traveller's point of view, which handbooks as a rule are careful to avoid, for fear of hurting the feelings of advertisers. Readers may feel assured of its impartiality and confide in its advice." As a matter of fact this independent attitude was the cause of several advertisements being withheld.

. ·. We regret that the reviewer used the words "each one" instead of the word several.—Ed. The Hospital.

